# Luminescence of Tb_3_Al_5_O_12_ phosphors co-doped with Ce^3+^/Gd^3+^ for white light-emitting diodes

**DOI:** 10.3762/bjnano.10.123

**Published:** 2019-06-14

**Authors:** Yu-Guo Yang, Lei Wei, Jian-Hua Xu, Hua-Jian Yu, Yan-Yan Hu, Hua-Di Zhang, Xu-Ping Wang, Bing Liu, Cong Zhang, Qing-Gang Li

**Affiliations:** 1 Advanced Materials Institute, Qilu University of Technology (Shandong Academy of Sciences), Jinan 250014, China; 2 Qilu University of Technology (Shandong Academy of Sciences), Advanced Materials Institute, Key Laboratory of Light Conversion Materials and Technology of Shandong Academy of Sciences, Jinan 250014, China; 3 Qilu University of Technology (Shandong Academy of Sciences), Advanced Materials Institute, Shandong Provincial Key Laboratory of High Strength Lightweight Metallic Materials, Jinan 250014, China

**Keywords:** luminescence, Tb_3_Al_5_O_12_:Ce^3+^/Gd^3+^, white light-emitting diodes (WLEDs)

## Abstract

Tb_2.96−_*_x_*Ce_0.04_Gd_x_Al_5_O_12_ phosphors were synthesized through solid-state reactions. The influence of Gd^3+^ on the luminescence was investigated. Under the excitation at 460 nm, Tb_2.96_Ce_0.04_Al_5_O_12_ shows the characteristic emission band of Ce^3+^ with a peak wavelength at about 554 nm. After co-doping Gd^3+^ into Tb_2.96_Ce_0.04_Al_5_O_12_, the peak wavelength of the Ce^3+^ emission band shifts to longer wavelengths, which is induced by the increasing crystal field splitting. However, the Ce^3+^ emission intensity also decreases because the substitution of Tb^3+^ with Gd^3+^ causes lattice deformation and generates numerous structural and chemical defects. By comparing the light parameters of white light-emitting diodes (WLEDs) containing Y_2.96_Ce_0.04_Al_5_O_12_, Tb_2.96_Ce_0.04_Al_5_O_12_ and Tb_2.81_Ce_0.04_Gd_0.15_Al_5_O_12_ phosphors, we can find that the WLED containing the Tb_2.81_Ce_0.04_Gd_0.15_Al_5_O_12_ phosphor generates warmer light than the WLEDs containing Y_2.96_Ce_0.04_Al_5_O_12_ and Tb_2.96_Ce_0.04_Al_5_O_12_ phosphors. Moreover, the WLEDs fabricated by integrating a blue LED chip and Ce^3+^/Gd^3+^-co-doped Tb_3_Al_5_O_12_ phosphors show outstanding colour stability when driven under different currents.

## Introduction

Currently, the most popular fabrication model of white light-emitting diodes (WLEDs) is to combine blue chips with yellow Y_3_Al_5_O_12_:Ce^3+^ phosphors, which has the disadvantages of low colour-rendering index (CRI) and high correlated colour temperature (CCT) [1–2]. At the same time, this type of WLEDs has the advantages of long lifetime, eco-friendliness, high luminous efficiency and low energy consumption, which helps to mitigate two serious issues in the world, namely ecological crisis and energy dilemma. As a result, various attempts have been made to address the shortcomings in this type of WLED. To date, two common methods are red-light compensation and the red-shift of Ce^3+^ emission band in Y_3_Al_5_O_12_. Red-light compensation is generally achieved by adding a red-emission phosphor, such as Eu^2+^-doped materials [3], materials doped with trivalent lanthanide ions (e.g., Eu^3+^ and Sm^3+^) [4–8], Mn^4+^-doped materials [9–12], or Ce^3+^/Cr^3+^-co-doped Y_3_Al_5_O_12_ [13–14]. The red-shift of the Ce^3+^ emission band in Y_3_Al1_5_O_12_ is achieved, in general, through ion substitution, such as Ca^2+^–Mg^2+^–Si^4+^ [15], Si^4+^–N^3−^ [16–17], Mg^2+^–Si^4+^/Ge^4+^ [18–20], or Gd^3+^ [21–22].

Tb_3_Al_5_O_12_ has a garnet structure similar to Y_3_Al_5_O_12_. A series of doped Tb_3_Al_5_O_12_ phosphors have been synthesized, such as Tb_3_Al_5_O_12_:Ce^3+^ [23–25], Tb_3_Al_5_O_12_:Ce^3+^/Eu^3+^ [26], Tb_3_Al_5_O_12_:Eu^3+^ [27], and Tb_3_Al_5_O_12_:Ce^3+^/Ga^3+^ [28]. The results show that Tb_3_Al_5_O_12_ is also a good host for various ions and the luminescent properties could be tuned by co-doping different ions into the Tb_3_Al_5_O_12_ host. The Tb_3_Al_5_O_12_:Ce^3+^ phosphor also shows a yellow emission band. But the emission wavelength is longer than that of the Y_3_Al_5_O_12_:Ce^3+^ phosphor because Tb^3+^ ions produce a stronger crystal field effect [23–25]. The longer emission wavelength of Tb_3_Al_5_O_12_:Ce^3+^ is more suitable for WLEDs used as indoor illumination than that of Y_3_Al_5_O_12_:Ce^3+^. It is known that the sensitivity of human eyes to red light decreases strongly as soon as the wavelength is longer than 611 nm [9]. We aimed to shift the emission wavelength of Tb_3_Al_5_O_12_:Ce^3+^ to a longer wavelength that is, however, still shorter than 611 nm. In this work, we report the synthesis and luminescence of a series of Ce^3+^/Gd^3+^-co-doped Tb_3_Al_5_O_12_ phosphors. The effect of co-doping Gd^3+^ on the luminescence of Tb_3_Al_5_O_12_:Ce^3+^ was investigated. It is found that the co-doped Gd^3+^ leads to a red-shift of the Tb_3_Al_5_O_12_:Ce^3+^ emission.

## Results and Discussion

**Figure 1:**
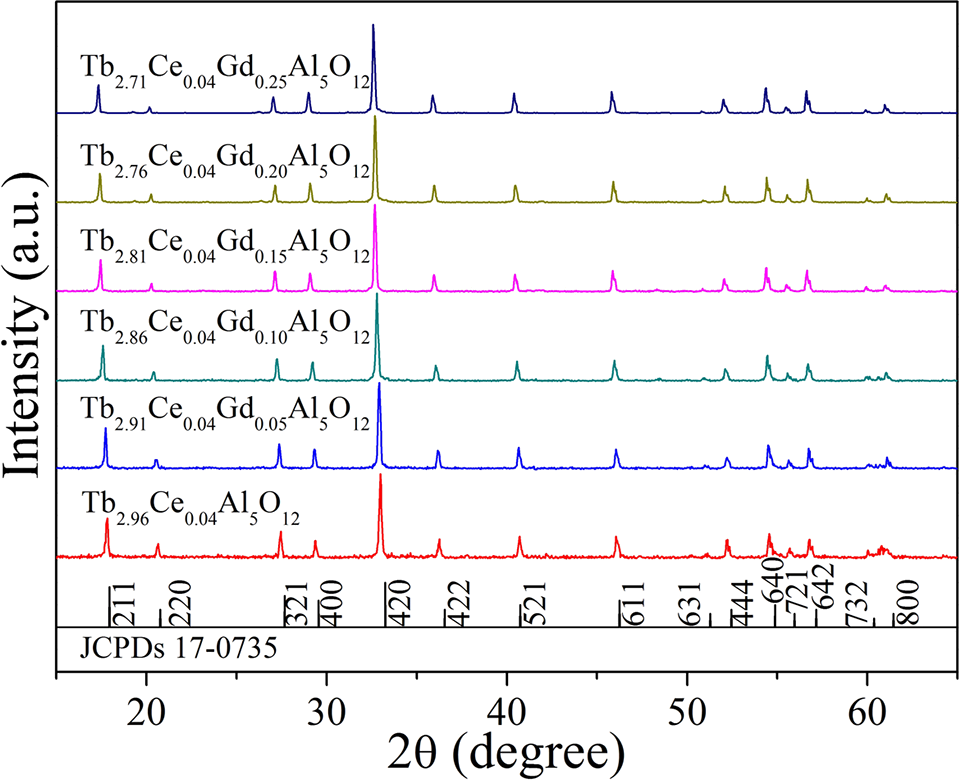
XRD patterns of Tb_2.96−_*_x_*Ce_0.04_Gd*_x_*Al_5_O_12_ phosphors.

The phase of the synthesized Tb_2.96-x_Ce_0.04_Gd_x_Al_5_O_12_ phosphors was confirmed by using XRD analysis. As shown in Figure 1, the diffraction peaks of Tb_2.96−_*_x_*Ce_0.04_Gd*_x_*Al_5_O_12_ (*x* = 0, 0.05, 0.10, 0.15, 0.20, and 0.25) phosphors are well in accordance with the JCPDs card no. 17-1735, meaning that Ce^3+^/Gd^3+^ ions have been doped into the Tb_3_Al_5_O_12_ host entirely and formed a solid solution. Moreover, the diffraction peaks shift to lower 2θ angles with increasing *x* values, which is induced by the substitution of Tb^3+^ with Ce^3+^/Gd^3+^. The ionic radii of Tb^3+^, Ce^3+^ and Gd^3+^ are 1.040 Å (CN = 8), 1.143 Å (CN = 8) and 1.053 Å (CN = 8), respectively. Due to the same valence and similar ionic radii of Tb^3+^, Ce^3+^, and Gd^3+^, Tb^3+^ ions are replaced by Ce^3+^ and Gd^3+^ ions in Ce^3+^/Gd^3+^ co-doped Tb_3_Al_5_O_12_ phosphors. The larger ionic radii of Ce^3+^ and Gd^3+^ lead to the increase of the cell volume, which induces the shifts to lower 2θ angles of the diffraction peaks.

**Figure 2:**
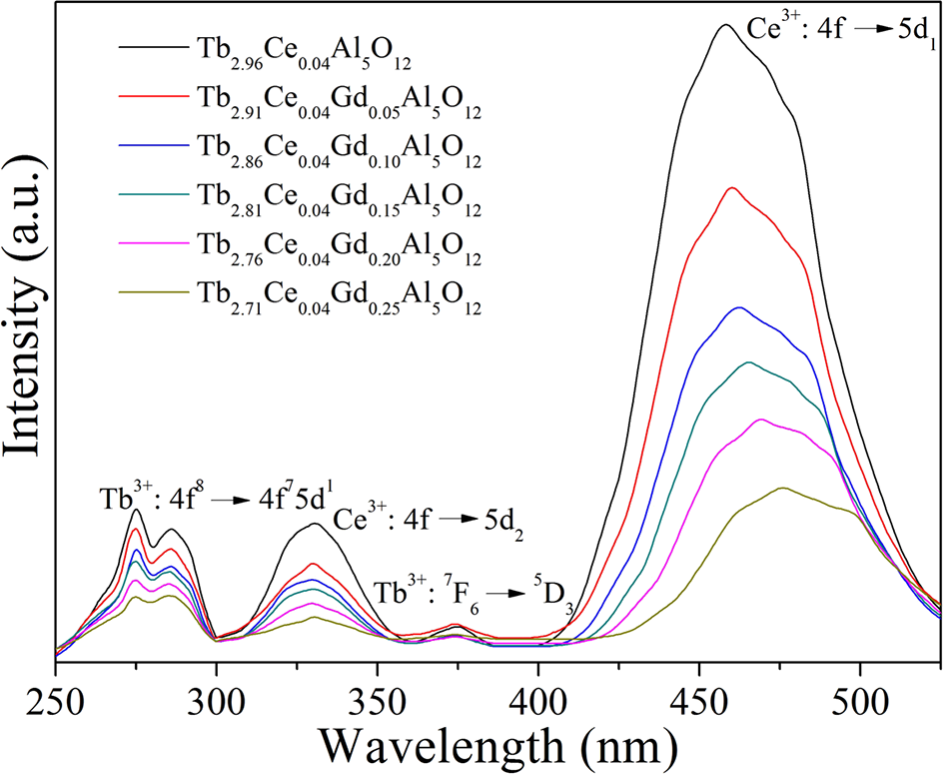
Excitation spectra of Tb_2.96−_*_x_*Ce_0.04_Gd*_x_*Al_5_O_12_ phosphors.

The excitation spectra of Tb_2.96−_*_x_*Ce_0.04_Gd*_x_*Al_5_O_12_ phosphors, which show the excitation bands of Tb^3+^ and Ce^3+^ ions, is given in Figure 2. The excitation bands in the range of 250–300 nm with two excitation peaks at 275 and 286 nm correspond to the 4f^8^→4f^7^5d^1^ inter-configurational transitions of Tb^3+^ [28]. The weak excitation band with a peak at 375 nm is induced by the ^7^F_6_→^5^D_3_ transition of Tb^3+^ [29]. Moreover, the 4f^8^→4f^7^5d^1^ transition of Tb^3+^ overlaps with the 4f→5d_2_ transition of Ce^3+^, which results in the excitation band with a peak at 331 nm [22,26]. The strongest excitation band with a peak at 457 nm corresponds to the 4f→5d_1_ transition of Ce^3+^ [22]. It can be seen from Figure 2 that the excitation band corresponding to the 4f→5d_1_ transition of Ce^3+^ shifts to shorter wavelengths gradually with the increase of *x*, which is induced by the splitting of the Ce^3+^ 5d state. The increasing Gd^3+^ concentration leads to an intensified crystal field, which results in a stronger of splitting of the Ce^3+^ 5d state. As a result, the 4f→5d_1_ transition of Ce^3+^ shifts to shorter wavelengths, but the 4f→5d_2_ transition of Ce^3+^ shifts to longer wavelength. Herein, the shift of the 4f→5d_2_ transition of Ce^3+^ to longer wavelengths cannot be seen clearly because of its overlaps with the 4f^8^→4f^7^5d^1^ transition of Tb^3+^.

**Figure 3:**
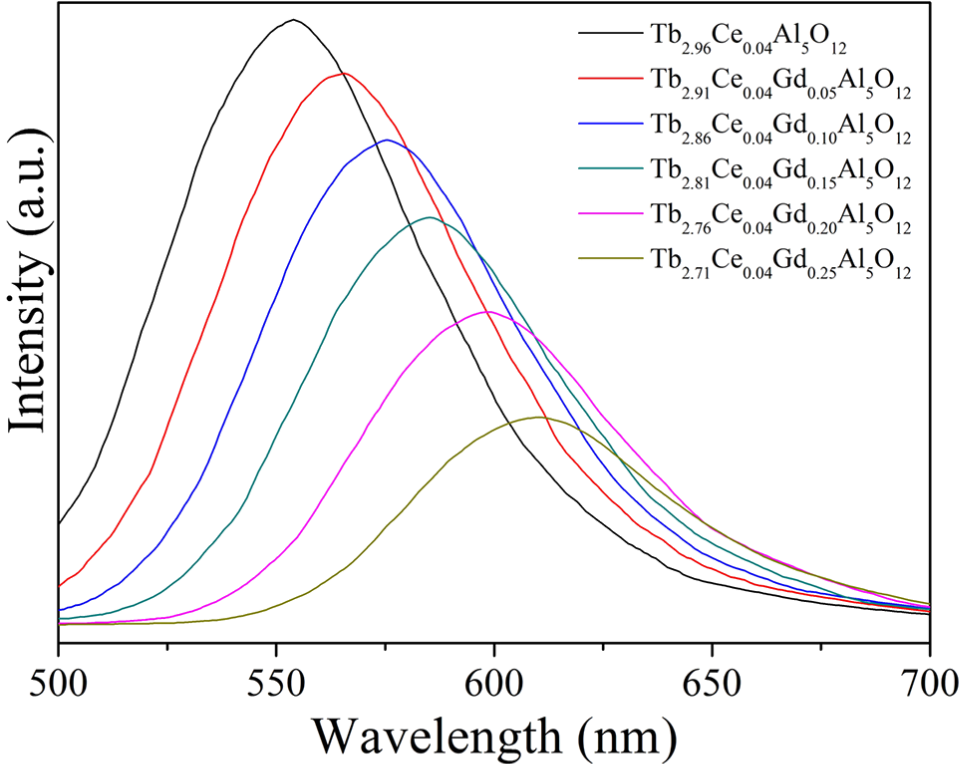
Emission spectra of Tb_2.96−_*_x_*Ce_0.04_Gd*_x_*Al_5_O_12_ phosphors.

**Figure 4:**
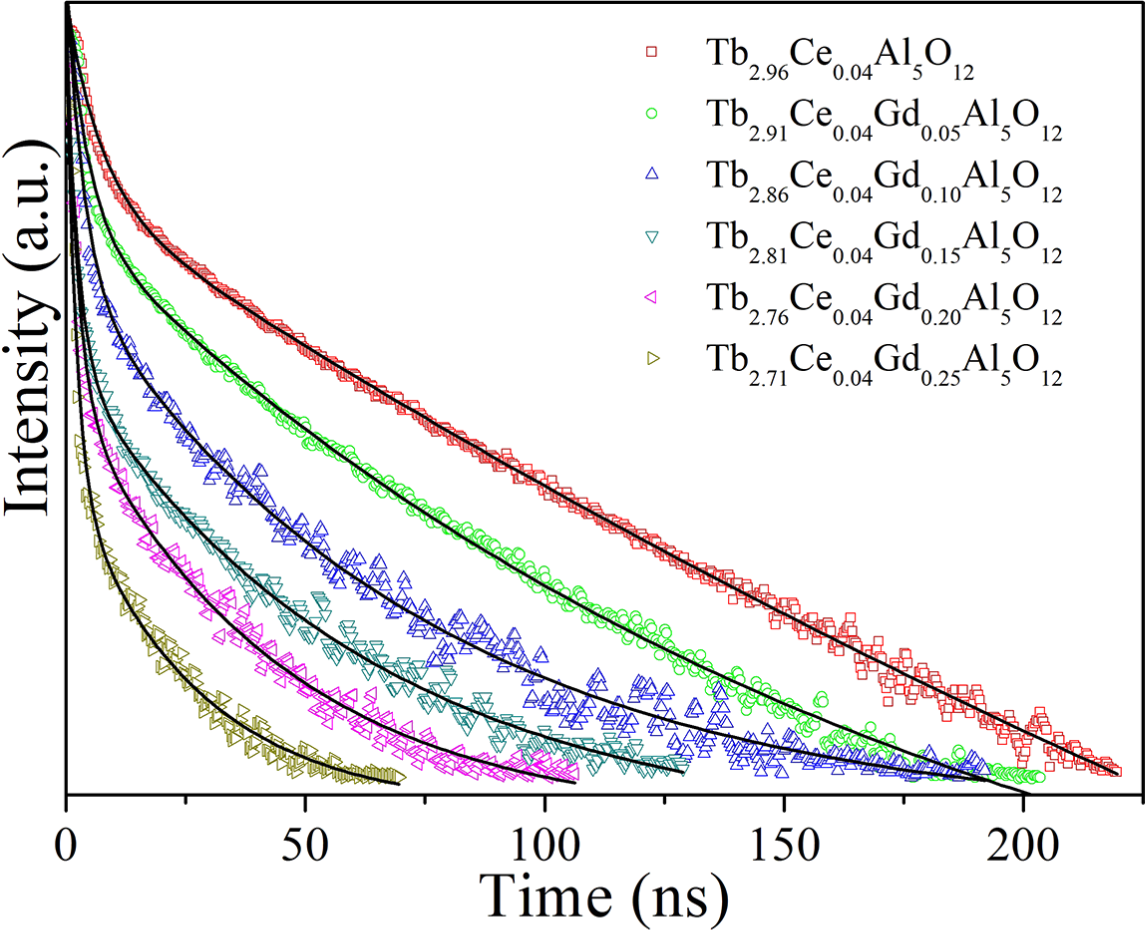
Decay curves of the Ce^3+^ emission from Tb_2.96−_*_x_*Ce_0.04_Gd*_x_*Al_5_O_12_ phosphors.

Under excitation at 460 nm, Tb_2.96−_*_x_*Ce_0.04_Gd*_x_*Al_5_O_12_ phosphors show the characteristic emission band of Ce^3+^, as shown in Figure 3. One feature is the red-shift of the Ce^3+^ emission with increasing Gd^3+^ concentration and the other is the decrease of emission intensity with increasing Gd^3+^ concentration. The emission band of the Tb_2.96_Ce_0.04_Al_5_O_12_ phosphor peaks at about 554 nm. For the Tb_2.71_Ce_0.04_Gd_0.25_Al_5_O_12_ phosphor, the peak wavelength of the emission band shifts to 610 nm. It is well known that the emission of Ce^3+^ depends on the crystal field splitting. The crystal field splitting can be calculated through the following equation:


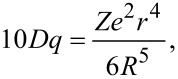


where 10*Dq* is the crystal field splitting parameter, *Z* is the anion charge, *e* is the electron charge, *r* is the radial distance of the d orbital from the nucleus, and *R* is the bond length [30–31]. Gd^3+^ has a larger ionic radius than of Tb^3+^. As a result, the Ce^3+^–O^2−^ bond length in Tb_3_Al_5_O_12_:Ce^3+^/Gd^3+^ decreases when Tb^3+^ ions are replaced by Gd^3+^ ions. The decrease of the Ce^3+^–O^2−^ bond length results in an increase of the crystal field splitting, which in turn leads to the red-shift of the Ce^3+^ emission. This result is in accordance with the excitation spectra. Moreover, the substitution of Tb^3+^ with Gd^3+^ causes lattice deformation and generates numerous structural and chemical defects, which results in a decrease of the Ce^3+^ emission intensity [32]. The decay characteristics of the synthesized Tb_2.96−_*_x_*Ce_0.04_Gd*_x_*Al_5_O_12_ phosphors were also investigated. Figure 4 gives the decay curves of Tb_2.96−_*_x_*Ce_0.04_Gd*_x_*Al_5_O_12_ phosphors. The decay curves of the Ce^3+^ emission fit well with the second-order exponential formula


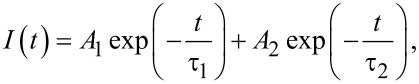


where *I* is the emission intensity, *A*_1_ and *A*_2_ are constants, *t* is the time, and τ_1_ and τ_2_ are the rapid and the slow lifetime, respectively. The average lifetime (τ*) can be calculated through


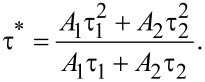


The calculated τ* values for Tb_2.96−_*_x_*Ce_0.04_Gd*_x_*Al_5_O_12_ with *x* = 0, 0.05, 0.10, 0.15, 0.20, and 0.25 are 35.23, 31.46, 28.52, 26.37, 23.58 and 19.45 ns, respectively.

**Figure 5:**
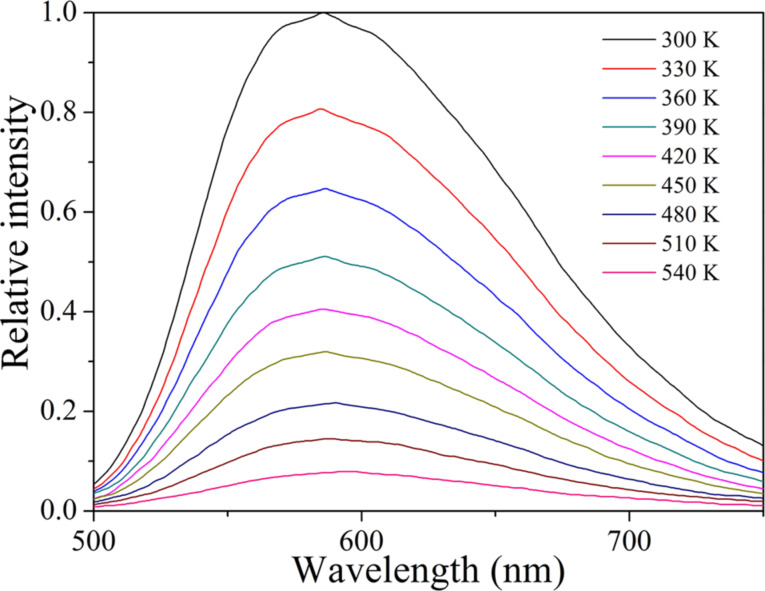
Emission spectra of Tb_2.81_Ce_0.04_Gd_0.15_Al_5_O_12_ at different temperatures.

The thermal stability of a phosphor is crucial for its applications in WLEDs. Thus, the emission spectra of a typical phosphor (Tb_2.81_Ce_0.04_Gd_0.15_Al_5_O_12_) at different temperatures were measured and the results are shown in Figure 5. The emission intensity decreases continuously with the increasing temperature in the range of 300–540 K. As the temperature increases from 300 to 390 K, the emission intensity decreases by about 49%. Photon interaction plays an important role in thermal quenching, in which emission centres are thermally activated and the energy is released through a nonradiative transition [31]. It is known that the probability of nonradiative transitions increases with increasing temperature. As a result, the emission intensity decreases with increasing temperature because of the higher number of nonradiative transitions.

**Figure 6:**
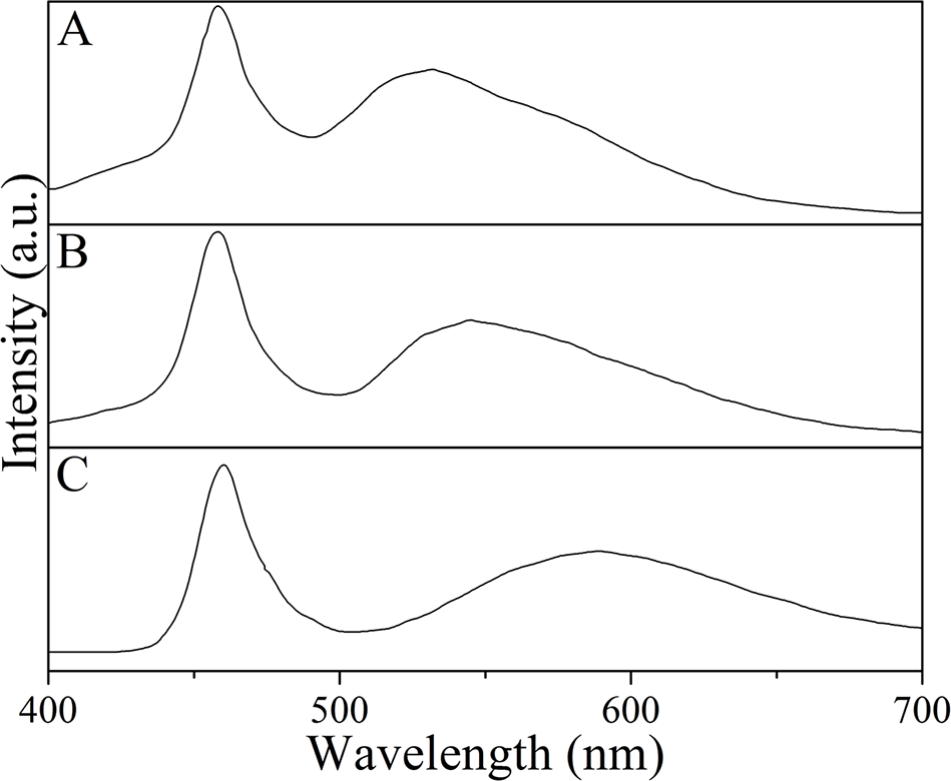
Electroluminescence spectra of WLEDs by combining blue LED chip with Y_2.96_Ce_0.04_Al_5_O_12_ (A), Tb_2.96_Ce_0.04_Al_5_O_12_ (B) and Tb_2.81_Ce_0.04_Gd_0.15_Al_5_O_12_ (C) phosphors.

WLEDs were fabricated by combining a blue LED chip (460 nm) with Y_2.96_Ce_0.04_Al_5_O_12_, Tb_2.96_Ce_0.04_Al_5_O_12_ and Tb_2.81_Ce_0.04_Gd_0.15_Al_5_O_12_ phosphors. The electroluminescence spectra under an operating current of 20 mA for the fabricated WLEDs are given in Figure 6. All of spectra consist of the blue excitation band of the LED chip and the emission band of the phosphor. The emission bands of the phosphors shift from 532 nm for Y_2.96_Ce_0.04_Al_5_O_12_ (Figure 6A) through 545 nm for Tb_2.96_Ce_0.04_Al_5_O_12_ (Figure 6B) to 589 nm for Tb_2.81_Ce_0.04_Gd_0.15_Al_5_O_12_ (Figure 6C). The CIE chromaticity coordinates for the light from these three WLEDs are (0.325, 0.349), (0.368, 0.351), and (0.376, 0.338), respectively. The CCT values of the light from these three WLEDs are 5828, 4158, and 3767, respectively. These results suggest that Tb_2.81_Ce_0.04_Gd_0.15_Al_5_O_12_ is a suitable phosphor for applications in WLEDs with low CCT for indoor lighting.

**Figure 7:**
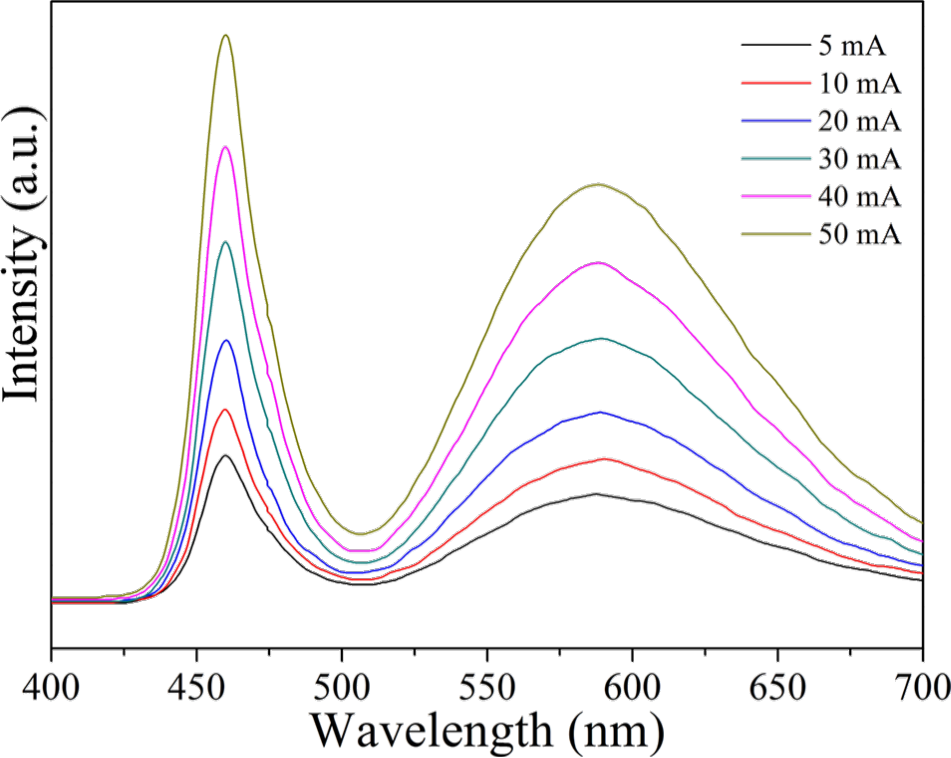
Electroluminescence spectra of a WLED with Tb_2.81_Ce_0.04_Gd_0.15_Al_5_O_12_ phsphor under different forward-bias currents.

Generally, the colour stability of a LED device can be examined through measuring the colour deviation under different driving currents [33]. Figure 7 shows the electroluminescence spectra of a WLED containing the Tb_2.81_Ce_0.04_Gd_0.15_Al_5_O_12_ phosphor under forward-bias currents of 5, 10, 20, 30, 40, and 50 mA. It can be seen that the intensity of the WLED increases with increasing current. Moreover, the shape and the peak of the bands corresponding to the LED chip and phosphor are consistent under different driving currents. This suggests the outstanding colour stability of the fabricated WLEDs.

## Conclusion

We synthesized a series of Tb_2.96−_*_x_*Ce_0.04_Gd*_x_*Al_5_O_12_ phosphors through solid-state reactions. The doping with Ce^3+^/Gd^3+^ ions does not lead to a phase change of the Tb_3_Al_5_O_12_ host but induces a slight increase of cell volume. Under excitation at 460 nm, the Tb_2.96_Ce_0.04_Al_5_O_12_ phosphor shows the characteristic emission band of Ce^3+^ with a peak wavelength of about 554 nm. The co-doped Gd^3+^ ions lead to a red-shift of the Ce^3+^ emission band and the red-shifts become larger with increasing Gd^3+^ concentration. Due to the larger ionic radius of Gd^3+^ compared with Tb^3+^, the substitution of Tb^3+^ with Gd^3+^ decreases the bond distance between Ce^3+^ and O^2−^, which leads to an increase of crystal field splitting. The increasing crystal field splitting induces the red-shift of the Ce^3+^ emission. The longer peak-wavelength of the Ce^3+^ emission for Gd^3+^ co-doped phosphors leads to a warmer light in WLEDs. The fabricated WLEDs by integrating a blue LED chip and the Ce^3+^/Gd^3+^ co-doped Tb_3_Al_5_O_12_ phosphors show outstanding colour stability when they are driven under different currents.

## Experimental

A series of Tb_2.96−_*_x_*Ce_0.04_Gd*_x_*Al_5_O_12_ (*x* = 0, 0.05, 0.10, 0.15, 0.20, and 0.25) phosphors were synthesized through solid-state reactions in a reduction atmosphere (5% H_2_/95% N_2_). Al_2_O_3_ (99.9%), Tb_4_O_7_ (99.9%), CeO_2_ (99.99%) and Gd_2_O_3_ (99.95%) were used as starting materials. For the purpose of decreasing the reaction temperature, 4 wt % H_3_BO_3_ (99.5%) was added as flux. In a typical synthesis, we firstly weighted the raw materials according to stoichiometric ratios. Then, the raw materials were mixed in an agate mortar by grinding for 30 min and the mixture was calcined at 1350 °C for 5 h in an alumina crucible. Finally, the product was collected and reground after the temperature decreased to room temperature.

The X-ray diffraction (XRD) measurements were performed on a Rigaku D/max-RA X-ray diffractometer using Cu Kα radiation (λ = 1.5406 Å) with the experimental parameters of 40 kV, 30 mA and 2°/min. The measurements of excitation, emission, temperature-dependence of emission and decay curves were carried out in an Edinburgh Instrument FLS920 spectrophotometer equipped with a 450 W xenon lamp as the excitation source. The measurements were spectrally corrected. The samples were heated to a certain temperature and kept at this temperature for 5 min by using a temperature controller. The rate of temperature increase was less than 1 °C/ min and the temperature deviation was less than 0.1 °C.
